# Retraction: High LINC01605 expression predicts poor prognosis and promotes tumor progression via upregulation of MMP9 in bladder cancer

**DOI:** 10.1042/BSR-20180562_RET

**Published:** 2021-04-06

**Keywords:** Bladder cancer, EMT, lncRNA LINC01605, Migration, MMP9

This Retraction follows an Expression of Concern relating to this article previously published by Portland Press.

This article is being retracted from Bioscience Reports at the request of the Editor-in-Chief and the Editorial Board following receipt of a notification from a reader, alerting the Editorial Board to two pairs of duplicated images, one in Figure 5C and the other in Figure 4C (previously corrected). The Editorial Office has also identified a third pair of duplicated images between Figures 4B and 5B.

The authors were contacted regarding the concerns raised and requested to correct the paper, however given the quantity of duplications within the article the Editorial Board stands by the decision to retract. The authors agree to the retraction.

